# Use of a Triple Prophylactic Strategy to Prevent Post-dural Puncture Headache: An Observational Study

**DOI:** 10.7759/cureus.7052

**Published:** 2020-02-20

**Authors:** Efrain Riveros Perez, Maria G Sanchez, Alexander Rocuts, Enoe Jimenez

**Affiliations:** 1 Anesthesiology, The Medical College of Georgia, Augusta University, Augusta, USA; 2 Anesthesiology and Perioperative Medicine, The Medical College of Georgia, Augusta University, Augusta, USA

**Keywords:** cosyntropin, epidural morphine, blood patch, obstetric anesthesia, post-dural puncture headache

## Abstract

Objective

Post-dural puncture headache (PDPH) after an accidental dural puncture is a very common complication of epidural analgesia/anesthesia. We observed the ability of a triple prophylactic method (epidural saline, morphine, and intravenous (IV) cosyntropin) to prevent PDPH and the need for a blood patch.

Methods

We retrospectively evaluated the effect of the combination of epidural saline, IV cosyntropin, and epidural morphine in parturients who had an accidental dural puncture with regard to the PDPH rate and the need for an epidural blood patch. We report a case series of patients with accidental dural puncture who underwent triple prophylaxis and other methods.

Results

Thirty-one patients were included in the study. Fourteen cases received triple prophylaxis (45%). Three patients in this group developed PDPH (21%), with two of them requiring a blood patch (14%). Nine patients underwent preventive measures other than triple prophylaxis with a PDPH rate of 55% and one needing a blood patch (11%). Conservative management was used in eight patients with PDPH and blood patch rates of 100% and 62%, respectively.

Conclusion

The triple prophylactic regimen of epidural saline, IV cosyntropin, and epidural morphine used after accidental dural puncture exhibits great potential to reduce the incidence of PDPH and the need for blood patch in obstetric patients.

## Introduction

Neuraxial techniques are widely used in obstetric practice to provide analgesia and anesthesia. A common complication associated with neuraxial anesthesia is post-dural puncture headache (PDPH), resulting from intentional or unintended puncture of the dura mater during the insertion of an epidural needle. The incidence of PDPH after spinal anesthesia is very low due to the widespread use of non-cutting small diameter spinal needles. The incidence of a “wet tap” (accidental dural puncture) during epidural injection has been reported to be 1.5%, with 52% - 85% of these patients developing PDPH [1-2].

When an obstetric patient develops PDPH, the institution of effective treatment is necessary. Although PDPH tends to resolve spontaneously over a couple of weeks, it carries the risk of potential complications [3]. PDPH interferes with the ability of the mother to take care of her baby, increases the risk of chronic headache, and limits early ambulation, thereby increasing the risk of venous thrombosis and pulmonary embolism [4-5]. As important as treating PDPH when it occurs is to prevent it when the dura is accidentally punctured during labor epidural placement. Multiple strategies have been reported in the medical literature to prevent this outcome. Preventive measures range from conservative strategies to invasive procedures. Bedrest and hydration have been traditionally recommended to try to prevent PDPH when a wet tap occurs; however, no conclusive evidence supports their use [6]. The use of oral and intravenous (IV) caffeine is insufficiently supported by clinical evidence [7]. Epidural morphine and IV cosyntropin (an ACTH analog) have been successfully used to prevent PDPH [8-9]. Epidural injection of normal saline reduces the gradient for cerebral spinal fluid (CSF) leak. The use of saline has shown variable results in different studies [10]. Although the results of some studies show insufficient evidence about its effectiveness, the administration of epidural saline is a technique relatively devoid of significant adverse effects.

In our institution, we recommend a multimodal approach to the prevention of PDPH after accidental dural puncture, based on strategies reported in the medical literature. The protocol consists of the immediate injection of epidural normal saline, accompanied by the administration of two medications immediately after delivery: IV cosyntropin and epidural morphine before catheter removal. We hypothesized that the multimodal protocol used at our institution (triple prophylaxis) after accidental wet tap (epidural saline, IV cosyntropin, and epidural morphine) has a favorable effect on the incidence of PDPH and reduces the need to use a blood patch.

## Materials and methods

After approval by the Institutional Review Board of Augusta University, we retrospectively studied obstetric patients who underwent labor epidural with a documented accidental dural puncture at our institution between January 2016 and November 2018. Patients with a history of migraines and chronic headaches, as well as those with neurologic disorders, were excluded. We collected demographic variables (e.g., age), morphometric variables (e.g., body mass index), obstetric information (e.g., gravidity and parity), prophylactic management to prevent PDPH, the presence and timing of post-procedure headache, and performance of epidural blood patch.

The multimodal approach protocol (triple prophylaxis) consists of the immediate transcatheter administration of 60 cc of epidural normal saline when the diagnosis of a wet tap is made, followed by 1 mg of intravenous cosyntropin, and 3 mg of epidural morphine before epidural catheter removal. A total of 2,700 medical records were reviewed to document the presence of a wet tap and prophylactic methods used to prevent PDPH, as well as the need for an epidural blood patch.

## Results

Thirty-two patients were identified to have an accidental dural puncture during epidural needle placement for labor or cesarean delivery. The incidence of a wet tap was 1.18%. The mean age was 28.65 ± 6.06. Demographic data are summarized in Table 1. Among the 32 patients who had a wet tap, 14 received triple prophylaxis, which represents 43.7% of the parturients who had a wet tap. Patients #17, 19, and 27 developed PDPH and only patients #17 and #27 decided to have a blood patch after the failure of conservative measures (14%). These measures included oral ibuprofen, oral acetaminophen, intravenous caffeine, and oral opioid-containing formulations. None of the patients who received triple prophylaxis required a second blood patch. These findings are described in Table 2.

**Table 1:** Demographic Information BMI: body mass index

Patient Number	Age	Gravidity	Parity	BMI
1	20	2	1	25.3
2	21	2	0	35.7
3	35	7	6	25.9
4	27	1	0	29.3
5	30	2	1	28.6
6	33	5	2	28.8
7	31	3	2	60.2
8	35	5	4	30.4
9	20	1	0	26.5
10	26	2	1	38.9
11	28	3	2	33.5
12	43	4	2	30.9
13	23	1	0	35.9
14	25	1	0	47
15	36	7	4	35.7
16	36	7	3	31.3
17	27	1	0	28.4
18	27	1	0	33.6
19	33	9	4	23.6
20	36	6	5	30.4
21	36	7	4	35.7
22	27	2	1	45.9
23	38	2	1	42
24	22	1	0	33.5
25	29	2	1	21.4
26	29	2	1	25.7
27	20	1	0	34.4
28	29	3	2	36
29	25	1	0	37.6
30	24	1	0	22.5
31	28	4	3	35.6
32	18	1	0	48.5

**Table 2:** Patients Who Received Triple Prophylaxis N: not performed; PDPH: post-dural puncture headache; Y: performed

Patient number	PDPH	Blood patch	Second blood patch
7	-	-	-
8	-	-	-
10	-	-	-
13	-	-	-
15	-	-	-
17	Y	Y	-
18	-	-	-
19	Y	-	-
21	-	-	-
23	-	-	-
24	-	-	-
27	Y	Y	-
28	-	-	-
29	-	-	-

Nine patients underwent preventive measures other than triple prophylaxis. Patients #5 and #31 received 1 mg of IV cosyntropin and 60 cc of epidural normal saline. Both parturients developed PDPH with only one patient (#31) needing a blood patch. Patients #9, #11, #26, and #32 received a combination of 1 mg IV cosyntropin and 3 mg of epidural morphine with only patient #26 developing PDPH and none requiring a blood patch. The epidural catheter was left intrathecally as a prophylactic method in patients #16 and #22. Both cases developed PDPH and were treated with a blood patch. Patient #25 received only 1 mg IV cosyntropin as prophylaxis and did not develop PDPH (Table 3). Eight patients who did not receive any prophylactic method after accidental dural puncture developed PDPH (100%). Five of those cases were treated with a blood patch (62%) (#1, #6, #12, #20, and #30). Only patient #12 in this group required a second blood patch (Table 4). Table 5 shows the frequency of preventive measures among patients who required a blood patch.

**Table 3:** Patient Who Received Other Prophylactic Methods NS: normal saline; PDPH: post-dural puncture headache; Y: procedure performed

Patient number	Prophylaxis	PDPH	Blood patch
5	Cosyntropin + epidural NS	Y	-
31	Cosyntropin + epidural NS	Y	Y
9	Cosyntropin + Duramorph	-	-
11	Cosyntropin + Duramorph	-	-
26	Cosyntropin + Duramorph	Y	-
32	Cosyntropin + Duramorph	-	-
16	Intrathecal catheter	Y	-
22	Intrathecal catheter	Y	-
25	Cosyntropin	-	-

**Table 4:** Patients Who Did Not Receive Prophylaxis PDPH: post-dural puncture headache; Y: procedure performed

Patient number	PDPH	Blood patch	Second blood patch
1	Y	Y	-
2	Y	-	-
3	Y	-	-
4	Y	-	-
6	Y	Y	-
12	Y	Y	Y
20	Y	Y	-
30	Y	Y	-

**Table 5:** Patients Who Had a Blood Patch and Prophylactic Method Used NS: normal saline

Patient number	Prophylaxis
1	None
6	None
12	None
17	Triple prophylaxis
20	None
27	Triple prophylaxis
30	None
31	Cosyntropin + epidural NS

## Discussion

Our case series shows that the administration of prophylactic triple prophylaxis (epidural normal saline, IV cosyntropin, and epidural morphine) has the potential to reduce both the incidence of PDPH and the need for blood patch treatment. In addition, it seems possible that the effect of individual therapies is synergistic when used in combination, as the incidence trends down along the spectrum from no therapy to the use of the three methods combined.

Invasive approaches have been tested as preventive measures against PDPH. Prophylactic epidural blood patch administration has been shown to be effective in isolated clinical trials with significant methodological flaws [10]. Since the administration of an epidural blood patch has potential risks, such as backache and (in rare instances) infection and neurologic deficit, it is not recommended at this point as a routine prophylactic practice for PDPH [11]. Placement of an intrathecal catheter after an accidental dural puncture has been proposed based on the rationale that it causes an inflammatory reaction that helps close the newly created dural orifice, thus limiting a CSF leak [11-12]. Systematic reviews have shown that an intrathecal catheter is unable to reduce the incidence of PDPH but decreases the need for an epidural blood patch. In our series, although two patients receiving this therapy developed PDPH, there was no need to perform a blood patch.

Inadvertent dural puncture during epidural placement occurs at a rate of 1.5% [1]. The incidence of PDPH after accidental dural puncture with a Tuohy needle may be as high as 76% - 85% [13]. In our case series, the incidence of PDPH when no prophylactic measure was instituted was 100%, which is high compared with most reports. This may be due to underreporting. In any case, the incidence is significantly reduced when triple prophylaxis was used. The effect of epidural morphine to prevent PDPH was evaluated by Al‐Metwalli in a clinical trial that included 50 parturients [8]. The authors used two doses of 3 mg of epidural morphine separated by a 24-hour period, finding PDPH rates of 12% and 48%, respectively. Hakim conducted a clinical trial in 90 parturients who had an accidental dural puncture to compare the use of 1 mg intravenous cosyntropin versus placebo [9]. The authors evidenced PDPH rates of 33.3% and 68.9% in the treatment and control group, respectively. Although the number of trials evaluating cosyntropin and epidural morphine is reduced, the quality of the studies is superior. Our case series used only one dose of epidural morphine, and this may have led to a suboptimal effect. Epidural saline, on the other hand, has been used to prevent PDPH, based on the assumption that it reduces the pressure gradients between the intrathecal and the epidural spaces, therefore, controlling the spinal fluid leak. Epidural infusion or repeat epidural bolus injections of saline after delivery reduced the incidence to 65% in a study that included 58 obstetric patients [14]. Trivedi et al. conducted a single-blinded clinical trial in 74 obstetric patients who had an accidental dural puncture [15]. In the group receiving between 40 and 60 cc of epidural saline, the PDPH rate was 66.7% compared to 87.5% in the group receiving conservative management. Despite the positive results of the aforementioned studies, a systematic review failed to demonstrate any prophylactic effects of epidural saline on PDPH incidence or the need for a blood patch (10). We propose that the triple prophylaxis potentiates the positive effects of individual methods by combining the analgesic effect of morphine, the pressure-equalizing effect of saline, and the increased production of cerebrospinal fluid and endorphin release induced by cosyntropin [16-17]. We may argue that even the small effect of epidural saline is maximized by the addition of the other two strategies. The result of the triple prophylaxis combination may be a significant reduction of PDPH incidence and the need for an epidural blood patch.

Our study has several limitations. The observational retrospective nature of the case series permits us to generate a hypothesis but impedes that we establish the effectiveness of the triple prophylaxis. Our intent was to simply observe whether the implementation of a triple prophylaxis method would add benefit to the prevention of PDPH, and most importantly, the need to perform an epidural blood patch, as it is clear from past experience and literature reports that an expectant approach after a dural puncture is insufficient. We did not compare different doses for our regimen, nor did we compare our cases with matched controls. Although we did not evidence any cases of respiratory depression, our case series did not evaluate the side effects of the medications employed. We identify significant opportunities for future research. Future evaluation of the triple prophylaxis is warranted to establish the effectiveness and magnitude of the effect on the prevention of PDPH. The safety profile of the triple prophylactic technique should also be investigated in depth.

## Conclusions

In conclusion, the triple prophylactic regimen of epidural saline, IV cosyntropin, and epidural morphine used after accidental dural puncture exhibits great potential to reduce the incidence of PDPH and the need for a blood patch in obstetric patients. The effect may be explained by the synergistic effect of different mechanisms of action targeting multiple levels in the complex pathophysiology of PDPH.
